# Antagonist of Growth Hormone-Releasing Hormone Receptor MIA-690 Suppresses the Growth of Androgen-Independent Prostate Cancers

**DOI:** 10.3390/ijms252011200

**Published:** 2024-10-18

**Authors:** Laura Muñoz-Moreno, M. Isabel Gómez-Calcerrada, M. Isabel Arenas, M. José Carmena, Juan C. Prieto, Andrew V. Schally, Ana M. Bajo

**Affiliations:** 1 Grupo de Investigación Cánceres de Origen Epitelial, Departamento de Biología de Sistemas, Campus Científico-Tecnológico, Universidad de Alcalá, 28805 Madrid, Spain; mi.gomez@edu.uah.es (M.I.G.-C.); mariajose.carmena@uah.es (M.J.C.); ana.bajo@uah.es (A.M.B.); 2 Unidad de Bioquímica y Biología Molecular, Departamento de Biología de Sistemas, Campus Científico-Tecnológico, Universidad de Alcalá, 28805 Madrid, Spain; 3 Unidad de Biología Celular, Departamento de Biomedicina y Biotecnología, Campus Científico-Tecnológico, Universidad de Alcalá, 28805 Madrid, Spain; 4 Endocrine, Polypeptide and Cancer Institute, Veterans Affairs Medical Center, Miami, FL 33125, USA; 5 Department of Pathology and Medicine, Division of Hematology/Oncology, Miller School of Medicine, University of Miami, Miami, FL 33136, USA; 6 Sylvester Comprehensive Cancer Center, Miller School of Medicine, University of Miami, Miami, FL 33136, USA

**Keywords:** GHRH-R antagonist, EGFR inhibitor, transactivation, prostate cancer, combination therapy

## Abstract

The development of resistance remains the primary challenge in treating castration-resistant prostate cancer (CRPC). GHRH receptors (GHRH-R), which are coupled to G-proteins (GPCRs), can mediate EGFR transactivation, offering an alternative pathway for tumour survival. This study aimed to evaluate the effects of the GHRH-R antagonist MIA-690, in combination with the EGFR inhibitor Gefitinib, on cell viability, adhesion, gelatinolytic activity, and the cell cycle in advanced prostate cancer PC-3 cells. The findings demonstrate a synergistic effect between MIA-690 and Gefitinib, leading to the inhibition of cell viability, adhesion, and metalloprotease activity. Cell cycle analysis suggests that both compounds induce cell cycle arrest, both individually and in combination. Furthermore, similar effects of the GHRH-R antagonist MIA-690 combined with Gefitinib were observed in PC-3 tumours developed by subcutaneous injection in athymic nude mice 36 days post-inoculation. These results indicate that combined therapy with a GHRH-R antagonist and an EGFR inhibitor exerts a stronger antitumor effect compared to monotherapy by preventing transactivation between EGFR and GHRH-R in CRPC.

## 1. Introduction

Cancer is one of the greatest challenges for various fields of medical research, as it is the second leading cause of death in society. According to estimates from GLOBOCAN, 19.3 million new cancer cases were diagnosed worldwide and 9.6 million cancer deaths occurred in 2020 [1]. Prostate cancer (PCa) is the second most common type of cancer and the second leading cause of cancer-related death worldwide [2,3]. Currently, localized PCa can be treated by radical prostatectomy with a good prognosis, as well as by androgen-deprivation therapy (ADT), which is very effective in castration-sensitive patients as androgen signalling is essential for prostate tumour cell growth [4]. However, despite initial response to this therapy, patients with advanced PCa eventually develop castration-resistant PCa (CRPC), leading to further tumour recurrence and progression, and current treatment options may be limited [5]. It is therefore necessary to better understand the underlying complexity of these types of cancer, by identifying new therapeutic targets susceptible to different drugs, and thus be able to expand treatment options in prostate cancer.

The role of the epidermal growth factor receptor (EGFR) family in cancer has been extensively investigated. Several studies have shown that these receptors are overexpressed in many solid tumours, such as breast, prostate, pancreatic, head and neck, ovarian, kidney and other cancers, and are also identified as biomarkers of tumour resistance. The EGFR family of transmembrane receptors consists of four members: EGFR/ HER1/erbB1, HER2/erbB2, HER3/erbB3, and HER4/erbB4; all of these belong to the class I receptor tyrosine kinase (RTK) family [6]. The physiological function of EGFRs is to regulate epithelial tissue homeostasis and development, but in a pathological setting, alterations in receptors of this family can lead to the development of cancer. Overexpression of EGFRs results in the stimulation of signalling pathways that lead to different processes important for tumour cells, such as proliferation, the inhibition of apoptosis, angiogenesis, migration, adhesion and invasion, and epithelial–mesenchymal transition (EMT). Thus, EGFRs and their signalling have been therapeutic targets for the development of new drugs in cancer treatment, ranging from monoclonal antibodies such as Panitumumab and Cetuximab to tyrosine kinase inhibitors (TKIs) such as Gefitinib and Erlotinib [7,8]. Gefitinib is currently approved for the treatment of non-small cell lung cancer [9]. Numerous studies have shown that Gefitinib inhibits the growth of xenografts of various human tumours, including prostate tumours, and potentiates the antitumour activity of other cytotoxic agents when used in combination [10,11,12,13]. However, the emergence of acquired resistance to Gefitinib has limited its usefulness as an antitumour agent, demonstrating the ability of these cells to overexpress alternative pathways and achieve a more aggressive phenotype. In addition, communication between EGFR family members and other receptors, such as G protein-coupled receptors (GPCRs), has been considered as a therapeutic tool in cancer patients [14,15].

Signalling mediated by growth hormone-releasing hormone (GHRH) is highly involved in tumour cell progression and metastasis [16]. Physiologically, GHRH is a peptide hormone secreted by the hypothalamus that regulates the synthesis and release of growth hormone (GH) in the anterior pituitary by binding to its receptor (GHRH-R), which belongs to the family of G protein-coupled receptors (GPCRs). Once released, GH stimulates hepatic secretion of insulin-like growth factor type I (IGF-I), which exerts negative feedback on the pituitary, decreasing GH release when necessary [16]. Several receptors for GHRH have been described, both the pituitary-type receptor (pGHRH-R) and four variants generated by alternative splicing (SV1–SV4), which are expressed physiologically in various human tissues, and also in several types of cancer, including breast and prostate cancer [16,17]. Both pGHRH-R and its variants are GPCRs and appear to mediate the effects of GHRH in tumours, where it acts as an autocrine/paracrine growth factor in several types of human cancers and, similarly, IGF-I acts as a potent mitogen for tumour cell growth and proliferation [18]. Various studies have shown that GHRH-R antagonists of the MIAMI series, such as MIA-602 and MIA-690, successfully reduce tumour cell proliferation as well as tumour growth of CRPC cell xenografts in animals [16,17,18,19,20]. These results suggest that GHRH-R antagonists show benefit in the treatment of CRPC, warranting further studies for the development of an effective therapy.

In addition to the canonical signalling pathways generated by GPCRs related to proliferation, differentiation, migration, and angiogenesis, GPCRs are also known to mediate the process of RTK transactivation, including EGFRs. This is a complex process that depends on several factors such as cell type, GPCR class, and the extracellular environment [21]. Two types of transactivation have been described, ligand-dependent or Triple-membrane-passing-signal (TMPS) and ligand-independent transactivation. In the first case, GPCR stimulation induces the activation of membrane-bound matrix metalloproteases (MMPs), such as the ADAM family, leading to the cleavage of RTK ligands and their release into the extracellular space [22]. These in turn bind to their receptors, such as EGFRs or other family members, promoting their dimerization and activation. Primarily, signalling pathways such as the Ras-ERK pathway and the PI3K-Akt pathway are activated. The second mechanism involves direct phosphorylation of the EGFR at the intracellular level, following stimulation of the GPCR by its ligand [14,23].

This is really important as the intercommunication between GPCR and EGFR provides another available pathway for the survival of tumour cells, including prostate tumour cells [18,19]. Thus, GHRH-R and EGFR could be therapeutic targets for antitumour drugs to block both signals. In CRPC, the combination of Gefitinib with other drugs has suppressed EGFR activity, resulting in a more pronounced decrease in cell viability compared to single treatment [24]. Therefore, it is interesting to consider combination therapies of EGFR- and GHRH-R-targeted drugs to improve the efficacy of antitumour treatment.

The aim of this study was to determine the effects of the GHRH-R antagonist MIA-690 in combination with the EGFR inhibitor Gefitinib on different cellular processes, such as viability, adhesion, and cell cycle, involved in cancer progression, using two in vitro experimental models and an androgen-independent prostate cancer cell line, PC-3.

## 2. Results

### 2.1. Effect of the GHRH-R Antagonist MIA-690 and the EGFR Inhibitor Gefitinib on Cell Viability in PC-3 Cells

**Figure 1:**
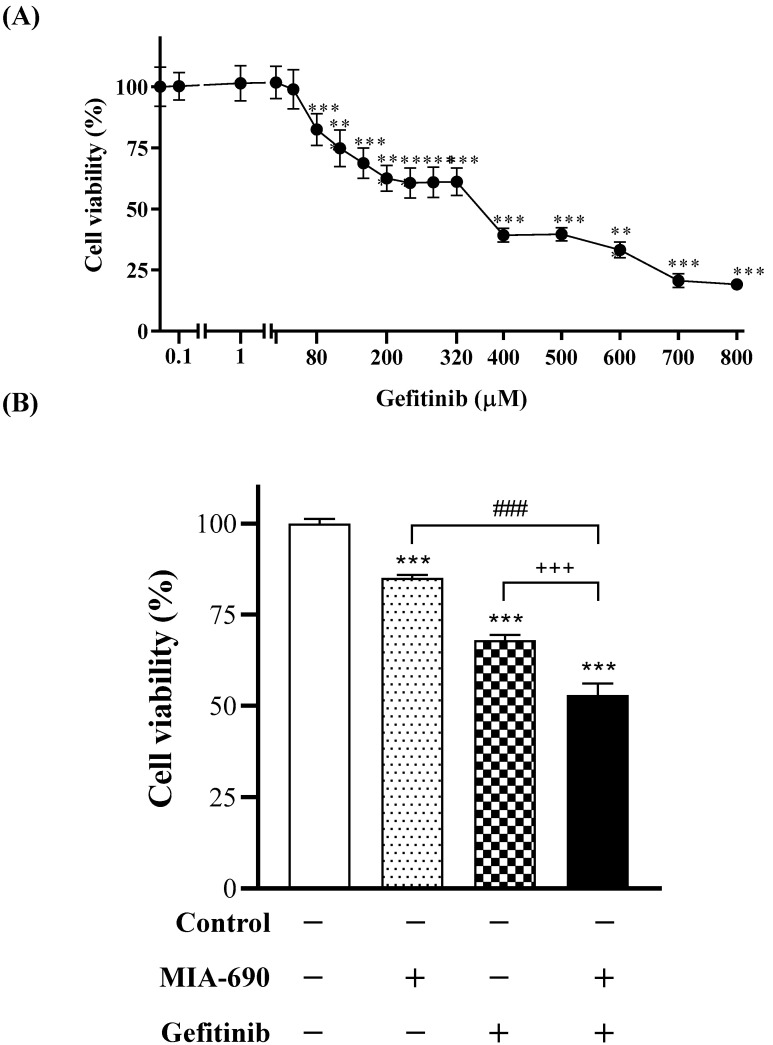
(**A**) Effect of EGFR inhibitor, Gefitinib, on cell viability in PC-3 cells. Outcome was evaluated by means of MTT assay. Results are expressed as percentage of control value. Data correspond to mean ± S.E.M. of at least two independent experiments in triplicate; **, *p* < 0.01; ***, *p* < 0.001 vs. control. (**B**) Effect of GHRH-R antagonist MIA-690 (0.1 μM) and Gefitinib (200 μM) on cell viability in PC-3 cells. Outcome was evaluated by means of MTT assay. Results are expressed as percentage of control value. Data correspond to mean ± S.E.M. of at least three independent experiments in triplicate; ***, *p* < 0.001 vs. control; ###, *p* < 0.001 vs. treatment with MIA-690 alone; +++, *p* < 0.001 vs. treatment with Gefitinib alone.

Initially, we aimed to define the mean inhibitory concentration (IC50) of Gefitinib in the advanced prostate cancer line, PC-3. For this purpose, cell viability assays were performed at different concentrations of Gefitinib (0.1–800 μM) during 24 h of treatment (Figure 1A). Cell viability decreases significantly with increasing Gefitinib concentration, in a dose-dependent manner. A concentration of 400 μM of Gefitinib or higher is required to achieve a decrease of more than 50% (60.7%). The study concentration of Gefitinib was 200 μM, resulting in a 40% reduction in cell viability, but without being excessively cytotoxic. The GHRH-R antagonist, MIA-690, was used at 0.1 μM, according to the group’s previous results [19].

Once the optimal concentration of Gefitinib was defined, PC-3 cell viability was studied for different treatment conditions at 24 h—control (no treatment), MIA-690 (0.1 μM), Gefitinib (200 µM), or the combination of both—in order to observe a possible synergistic effect (Figure 1B). Viability decreases significantly after treatment with MIA-690, Gefitinib, or both compared to the control. Significant differences are shown when comparing combination therapy to individual treatment with MIA-690 and Gefitinib. A decrease of 15% and 32% in the viability was observed with MIA-690 and Gefitinib, respectively, while the combination of both showed a 47.09% reduction compared to the control.

### 2.2. Effect of the GHRH-R Antagonist MIA-690 and the EGFR Inhibitor Gefitinib on Cell Adhesion in PC-3 Cells

**Figure 2:**
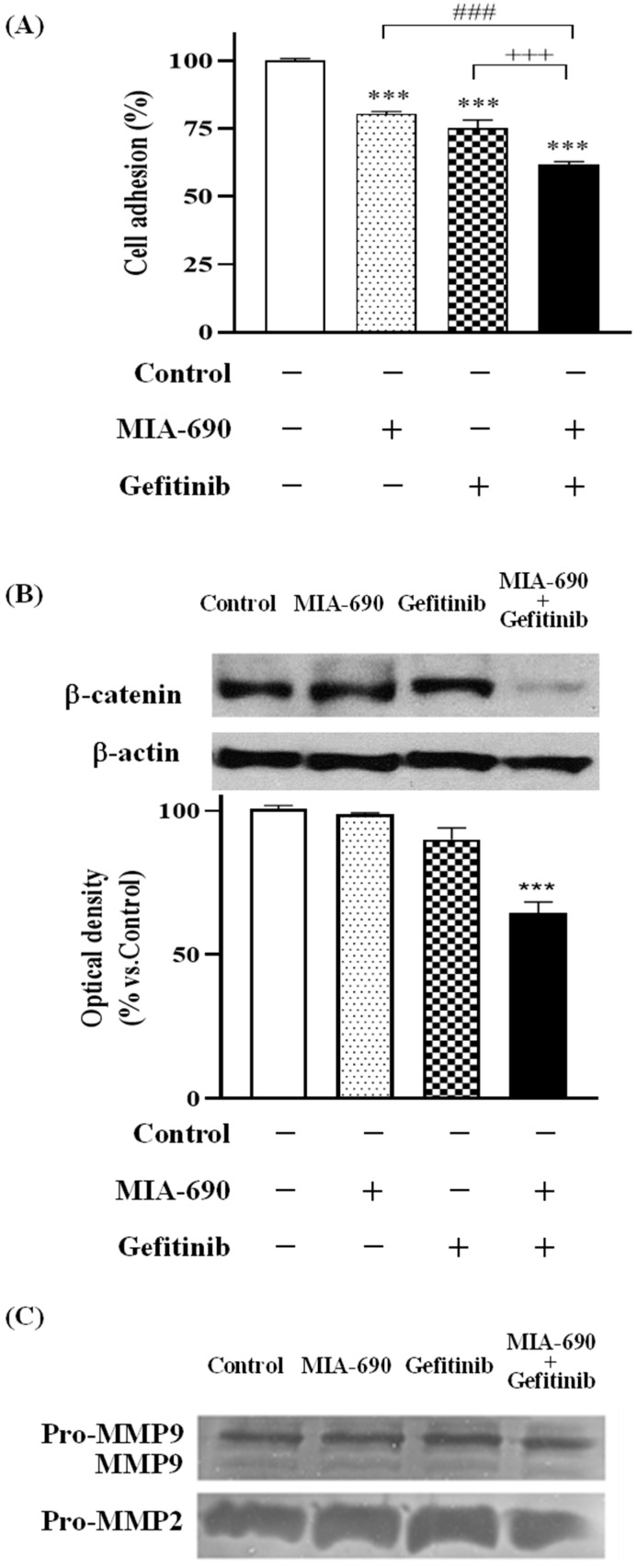
Effect of GHRH-R antagonist MIA-690 (0.1 μM) and Gefitinib (200 μM) on (**A**) cell adhesion to collagen in PC-3 cells. Results are expressed as percentage of control value at 0 and 60 min. Data correspond to mean ± S.E.M. of at least three independent experiments in triplicate; ***, *p* < 0.001 vs. control; ###, *p* < 0.001 vs. treatment with MIA-690 alone; +++, *p* < 0.001 vs. treatment with Gefitinib alone. (**B**) β-catenin levels in PC-3 cells by means of Western blot assay with specific antibody. A representative image is shown of at least two independent experiments. (**C**) Secretion of metalloproteinases (MMPs) 2 and 9 from PC-3 cells by means of Zimography assay. Both gelatinases are detected in their active (MMP-9 and MMP-2) and latent (pro-MMP-9 and pro-MMP-2) forms. The image of at least two independent experiments is shown.

To study adhesion in PC-3 cells, a collagen adhesion assay was performed at times 0 and 60 min. The time was chosen based on the group’s previous studies performed with PC-3 cells [19]. Figure 2A shows the effect of the GHRH-R antagonist, EGFR inhibitor, or a combination of both after 30 min of treatment. At time 0 min, there were no significant differences with respect to the control or between the different treatments, but at time 60 min there was a significant decrease in adhesion after treatment with 0.1 μM MIA-690 (19.62%), 200 μM Gefitinib (24.96%), and also in the combination of both with respect to the control (38.23%). In addition, the combined treatment of both compounds produced a significant reduction compared to the individual treatments.

### 2.3. Effect of the GHRH-R Antagonist MIA-690 and the EGFR Inhibitor Gefitinib on β-Catenin Expression in PC-3 Cells

Adhesion is one of the tumour processes involved in the initiation of metastasis, related to the extracellular matrix (ECM) degradation capacity of tumour cells. Cell–cell adhesion is controlled by proteins such as β-catenin. Therefore, β-catenin levels were assessed by Western blotting in prostate tumour cells after different treatments with the GHRH-R antagonist and the EGFR inhibitor, individually or in combination. Figure 2B shows the reduction in β-catenin levels in PC-3 cells by 98.2%, 90.25%, and 64.54% in the MIA-690, Gefitinib, and combined therapy groups, respectively, compared to the control.

### 2.4. Effect of the GHRH-R Antagonist MIA-690 and the EGFR Inhibitor Gefitinib on Metalloprotease Expression in PC-3 Cells

Metalloproteases are enzymes responsible for degrading the extracellular matrix (ECM), which favours the dissemination of tumour cells and the initiation of the metastasis process. In this case, the gelatinolytic activity of metalloproteases 2 (MMP2) and 9 (MMP9) is assessed by zymography, as they are the MMPs most involved in the progression of prostate cancer. Figure 2C shows the results after 24 h of treatment with the different compounds. The MMP9 levels were reduced by 15% and 12% in the MIA-690 and Gefitinib treatments, respectively. No differences are showed in pro-MMP9 and pro-MMP2 levels following treatment with MIA-690 and Gefitinib. The most important differences were observed in the reduction in pro-MMP9 by 30%, MMP9 by 63%, and pro-MMP2 by 48% in the combined treatment, compared to the control.

### 2.5. Effect of the GHRH-R Antagonist MIA-690 and the EGFR Inhibitor Gefitinib on the Cell Cycle in PC-3 Cells

The effect of the GHRH-R antagonist and EGFR inhibitor on the cell cycle of PC-3 was studied by flux cytometry. In the DNA histograms, several peaks can be observed corresponding to cells in the G0/G1 phase and cells in the G2/M phase, which have twice as much DNA (4n) as the population in G0/G1 (2n). Between both peaks are the S-phase cells, which have an intermediate DNA content, and it is also possible to observe a population prior to the G0/G1 peak, called the sub-G0 population, which refers to apoptotic cells as they show a reduction in their DNA content. 

**Figure 3:**
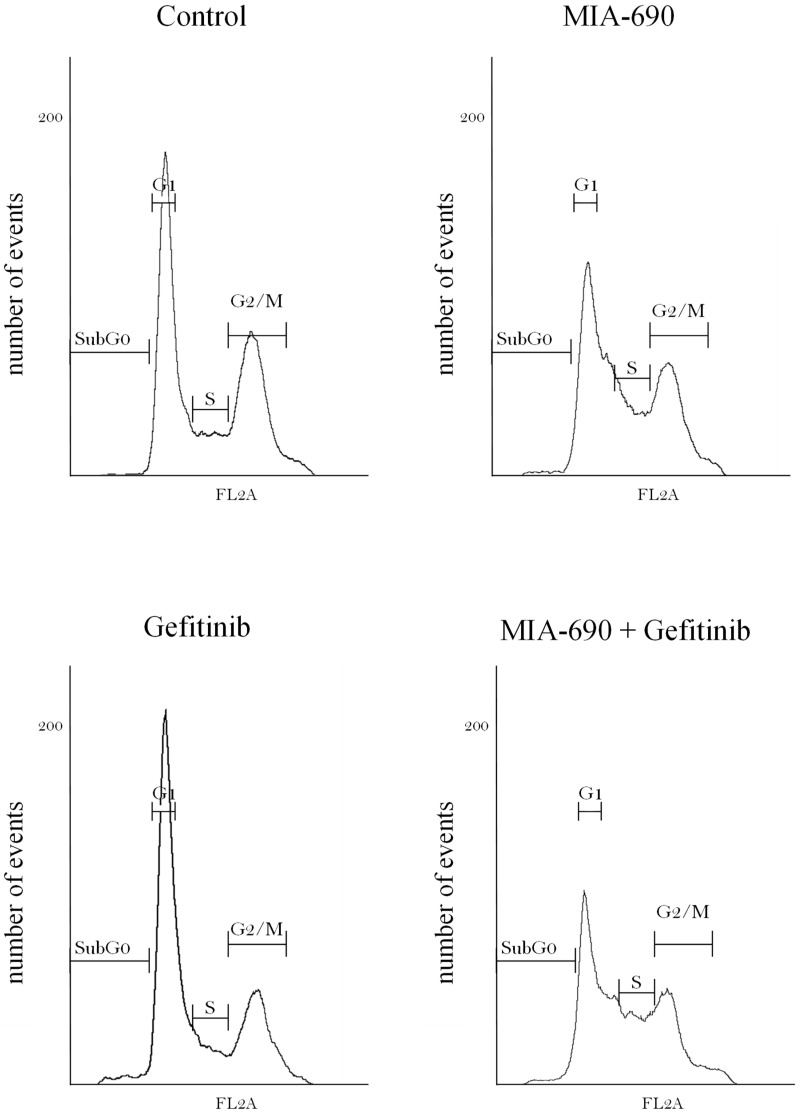
Effect of GHRH-R antagonist MIA-690 (0.1 μM) and Gefitinib (200 μM) on cell cycle in PC-3 cells by means of flow cytometry analysis. Histograms with DNA information in each phase of the cell cycle are shown: G0/G1 phase, S phase, and G2/M phase in control and 24 h-treated group. The image is shown of at least one independent experiment.

The results shows that MIA-690 treatment caused a 19% increase in S-phase cells, and an 8% and 14% reduction in G0/G1- and G2/M-phase cells, respectively, compared to the control (Figure 3). Gefitinib provoked a 7% accumulation of cells in the G0/G1 phase. However, the combination of the two produced a marked 24% reduction in cells in the G0/G1 phase and a 17% reduction in the G2/M phase, compared to control, as well as an increase in PC-3 cells in the S phase.

### 2.6. Effect of the GHRH-R Antagonist MIA-690 and the EGFR Inhibitor Gefitinib on the Growth of Xenografted PC-3 Human Prostate Cancer Cells

**Figure 4:**
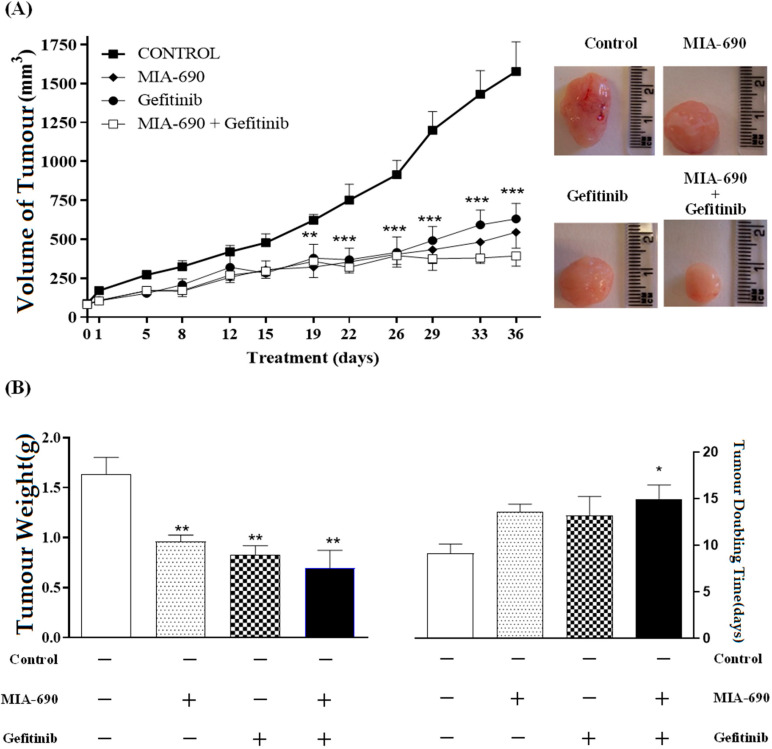
Effect of the GHRH-R antagonist MIA-690 (5 μg/day), Gefitinib (100 mg/Kg/day), and their combination on (**A**) the growth of s.c. PC-3 human androgen-independent prostate cancer in nude mice (nine mice per group). Treatment was started when tumours had grown to ∼85 mm^3^ and lasted for 36 days. The data in each bar show the means ± S.E.M. **, *p* < 0.01; ***, *p* < 0.001 vs. control (6 mice). Representative image of the tumours after treatment without and with corresponding compounds; (**B**) tumour weight and tumour doubling time. *, *p* < 0.05 vs. control; **, *p* < 0.01 vs. control. The weight of the mice, in all treatment groups, decreased by up to 10% throughout the experiment.

Treatment with MIA-690 and Gefitinib was performed after the injection of PC-3 cells in nude mice and further formation of tumours. Final tumour volume measurements revealed that MIA-690 significantly inhibited tumour growth by 65% (545.3 ±102 mm^3^) and Gefitinib by 61% (631.5 ± 98 mm^3^) after 36 days of treatment, as compared with the control group, which measured 1578.2 ± 190 mm^3^ (Figure 4A). Additionally, combined treatment with MIA-690 and Gefitinib resulted in the greatest inhibitory effect and diminished the growth of PC-3 tumours by 75% versus controls. Combined treatment with GHRH-R antagonist and EGFR inhibitor significantly extended tumour doubling time from 9.12+/−1.01 days to 14.97+/−1.5 days and provoked a 57.5% decrease in tumour weight as compared to controls (Figure 4B). At the end of the experiment, no significant differences in body weights were observed between groups, indicating that treatment with GHRH antagonists was not toxic to tumour-bearing animals.

### 2.7. Effect of the GHRH-R Antagonist MIA-690 and the EGFR Inhibitor Gefitinib on the Metastatic Potential of PC-3 Cells

**Figure 5:**
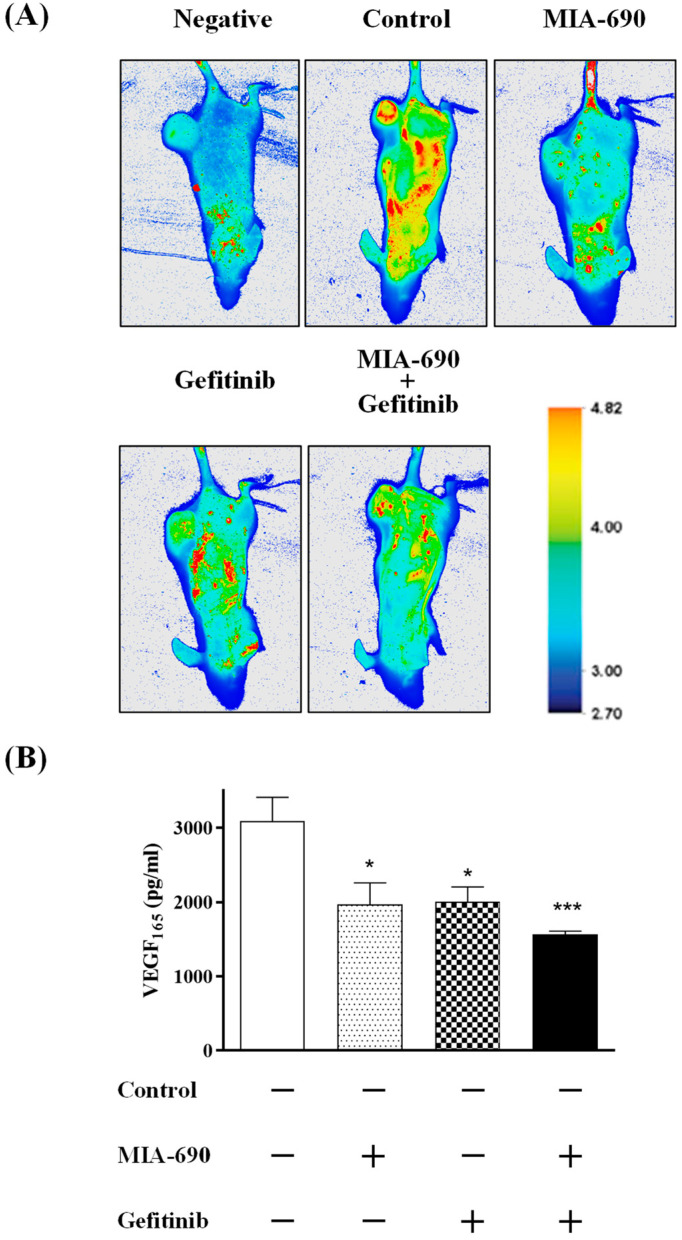
The antimetastatic potential of the GHRH-R antagonist (5 μg/day) and Gefitinib (100 mg/Kg/day) was evaluated by retention of IRDye 800CW 2-DeoxiGlucose (LI-COR) to tumour cells in nude mice. (**A**) The fluorescent contrast agent (15 nmol) was injected intravenously through the tail vein. Images were obtained and analyzed with an Odyssey Imaging System (LI-COR). As autofluorescence control, a mouse without dye was scanned. Images are representative from each group; (**B**) VEGF expression. The immunoexpression of VEGF_165_ was assessed from PC-3 tumours. Data in each bar are the means ± S.E.M. *, *p* < 0.05; ***, *p* < 0.001 vs. control.

An established characteristic of many cancer cells is an elevated rate metabolism; specifically, it is known that they have very active glucose metabolism. The antimetastatic potential of the GHRH-R antagonist and Gefitinib was evaluated by the retention of IRDye 800CW 2-DG to tumour cells. To further confirm the accumulation of such a probe, fluorescence in tumour and organs was detected 48 h after injection of the 2-DG. Inspection of the scanned mouse images revealed an increased glycolytic activity that was localized in several foci throughout the animal body (Figure 5A). Treated groups with MIA-690, Gefitinib, or both compounds show a low intensity of glycolysis activity in small foci.

### 2.8. Effect of the GHRH-R Antagonist MIA-690 and the EGFR Inhibitor Gefitinib on the Expression of Proangiogenic Factor VEGF

We observed in the tumour extraction process that tumour masses exhibited less blood supply than control animals after treatment with all compounds. To determine whether these tumours presented increased angiogenesis and its possible variations, we checked VEGF levels with an ELISA assay. VEGF expression showed a significant decrease of 26–27% in groups treated with GHRH antagonist MIA-690 or Gefitinib. The combination of both showed a 50% reduction in VEGF levels compared to the control (Figure 5B). 

### 2.9. Effect of the GHRH-R Antagonist MIA-690 and the EGFR Inhibitor Gefitinib on the Expression of GHRH, GHRH-R, Phosphorylated EGFR, MMP-2, and MMP-9

**Figure 6:**
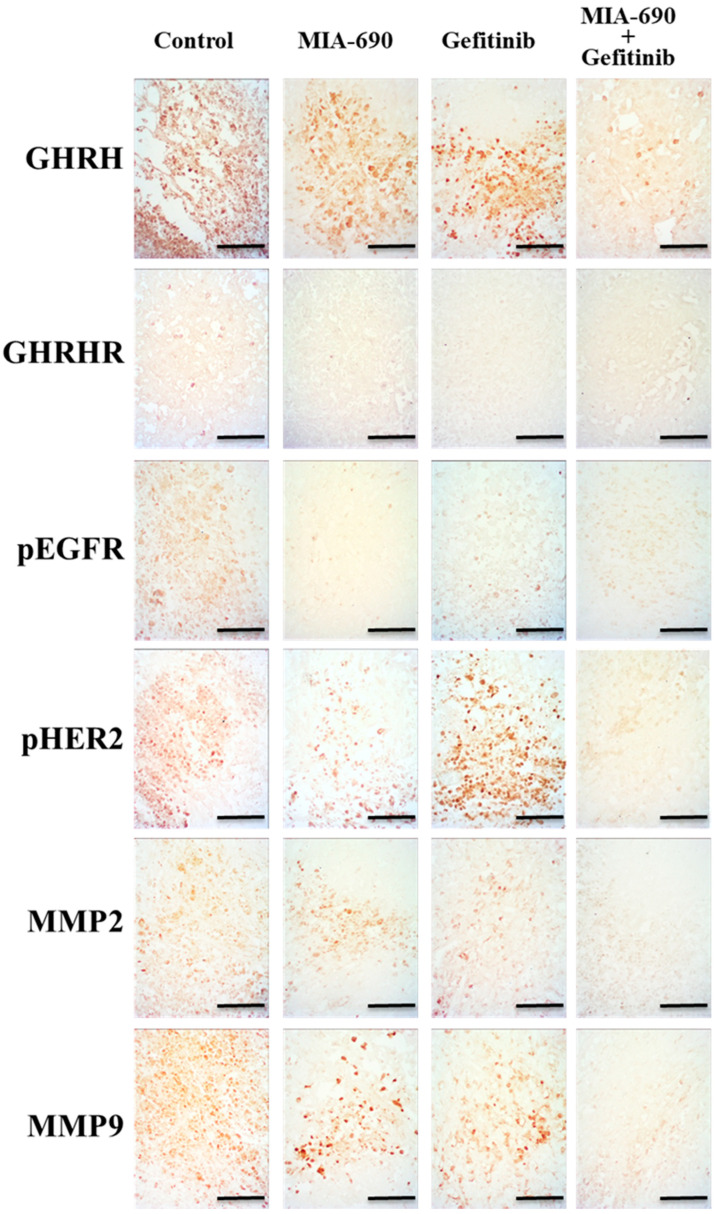
Effect of the GHRH-R antagonist MIA-690 (5 μg/day), Gefitinib (100 mg/Kg/day), and their combination on the expression of GHRH, GHRH-R, pEGFR, pHER2, MMP2, and MMP9. The immunoexpression was observed in histological sections from PC-3 tumours. For all figures, original magnification scale bar is 30 μm.

Once the in vivo experiment was performed, we studied the expression of different molecules involved in the processes of tumourigenesis, angiogenesis, and metastasis by means of immunohistochemistry (Figure 6). The results of the expression of the protumour factor GHRH in PC-3 tumours showed that after treatment with the GHRH-R antagonist and the EGFR inhibitor, there was a decrease in expression. With the combined treatment of both, the signal was much weaker. When we analyzed the expression of GHRH receptors in PC-3 tumours, we observed that treatment with the antagonist and the inhibitor caused very weak labelling of GHRH-R, which was non-existent in the samples from the combined treatment. Phosphorylated EGFR expression was decreased in treatments with Gefitinib and in the combination of both compounds.

The study of the immunodetection of metalloproteases in PC-3 tumours revealed that the combined treatment caused a decrease in MMP-2 and MMP-9; in the latter case, this was similar, although somewhat weaker, to that obtained after treatment with MIA-690.

## 3. Discussion

Effective treatment of patients with CRPC requires, at present, a search for new therapeutic targets. In this project, we evaluated the effect of the GHRH-R antagonist MIA-690 in combination with the EGFR inhibitor Gefitinib on different tumour processes in PC-3 cells. As discussed, the effect of GHRH-R antagonists and EGFR inhibitors has been shown to reduce cell viability and tumour growth of xenografts of different tumour types, including prostatic [13,17,20]. However, the emergence of resistance has led to the search for new alternatives such as combination therapies [8]. To improve the cytotoxicity of the GHRH-R antagonist MIA-690 and the EGFR inhibitor Gefitinib, the combination of both drugs has been proposed to block the intercommunication between GHRH-R and EGFR in the transactivation process [19]. 

Several researchers have evaluated the inhibitory effect of GHRH antagonists on CRPC cell viability [16,19,20]. Treatment with JMR-132, a pre-MIA series GHRH-R antagonist, in PC-3 cells showed a significant reduction in viability compared to control, according to the authors [16]. Similarly, our group showed that viability was reduced by 27–44% after MIA-690 treatment in PC-3 cells in a dose-dependent manner [19]. Other studies performed xenografts of CRPC cells in mice and observed a reduction in tumour volume after treatment with different GHRH antagonists of the MIA series [20]. Our results confirm previous studies, as MIA-690 caused a 15% reduction in viability compared to control in PC-3 cells. Moreover, one study confirmed that in PC-3 cells the combined administration of Gefitinib and Luteolin had a greater inhibitory effect on cell viability compared to single treatments [23]. In our study, the combination of MIA-690 and Gefitinib induced a more pronounced decrease in cell viability in the PC-3 cell line, which could indicate a synergistic effect between the GHRH-R antagonist and the EGFR inhibitor.

The initiation of the metastatic process is complex and involves numerous events, such as proliferation, the separation of cells from the primary tumour, adhesion in a new location, and migration or degradation of the extracellular matrix (ECM), among others. Increased adhesion to the ECM is considered an important step in the acquisition of metastatic properties in tumour cells. Therefore, some authors studied the effect of GHRH antagonists on cell adhesion, observing a 34% reduction compared to control following treatment with MIA-602 in breast cancer, glioblastoma, and ovarian cancer cells [24]. In our study, MIA-690 treatment also reduced adhesion in prostate cancer cells, but this effect was more pronounced when a combined therapy of MIA-690 with Gefitinib was used. This may be due to reduced adhesion to collagen (which mimics the ECM), or to the toxicity effect of treatment with both drugs in combination, which would lead to increased cell death and, thus, reduced adhesion. In addition to alterations in cell adhesion, the release of β-catenin from adherens junctions between cells is associated with the loss of cell–cell junctions and increased expression of target genes that promote tumour progression, such as *CD44*, *cyclin D*, and *c-Myc*. These molecules govern processes such as cell cycle regulation, cell adhesion, and proliferation [19]. Moreover, the inhibition of androgen activity results in an upregulation of the Wnt/β-catenin pathway, which subsequently promotes the androgen-independent growth of prostate cancer cells. In the absence of the androgen receptor, β-catenin is recruited to TCF (T-cell factor/lymphoid enhancer factor binding sites) binding sites, leading to enhanced transcription and, consequently, increased cellular proliferation [25,26]. Some groups have shown a reduction in β-catenin levels after treatment with MIA series GHRH antagonists in CRPC cells [19,24]. In our case, a preliminary study of β-catenin expression in PC-3 cells showed a reduced presence of this protein after combined treatment with MIA-690 and Gefitinib. This protein plays a significant role in the Wnt signalling cascade. Upon release from the cadherin complex, β-catenin functions as a transcriptional regulator of numerous target genes, including CD44, cyclin D, and c-Myc. These molecules are involved in tumour progression, governing processes such as cell cycle regulation, cell adhesion, and proliferation.

In the process of metastasis, ECM degradation plays a relevant role and is mediated by the activity of proteolytic enzymes, such as metalloproteases (MMPs), mainly MMP2 and MMP9. In addition to its direct proteolytic effects on the extracellular matrix (ECM), MMP-2 also activates other cellular substrates, such as FGFR and MMP-9, through enzymatic cleavage. MMP-9 plays a role in the regulation of angiogenesis; antisense suppression of MMP-9 expression in DU-145 and PC-3 cells resulted in a simultaneous inhibition of the gene expression of proangiogenic factors, including vascular endothelial growth factor (VEGF) and intercellular adhesion molecule-1 (ICAM-1) [27]. To study the effect of MIA-690 and Gefitinib on CRPC metastasis, MMP2 and MMP9 activity was investigated by zymography assays. Control cells showed higher activity, while the combination therapy decreased the activity of both pro- and active MMPs. Other studies also show similar results with GHRH antagonist treatment in prostate cancer cells [16,19,24], associating reduced MMP2 and 9 activity with reduced tumour cell motility and invasiveness. Furthermore, it has been shown that β-catenin-mediated signalling can regulate MMP9 expression, as may be occurring in our system. 

Our research on CRPC was complemented by preliminary cell cycle studies. The results seem to show an accumulation of S-phase cells after treatment with MIA-690 and Gefitinib. This could explain the decrease in viability previously observed with the combined treatment as a cycle arrest may be occurring. Other studies have shown a similar effect on the cell cycle in prostate cancer after treatment with GHRH antagonists [19]. 

Therefore, this study seems to point to a possible synergistic effect of the GHRH-R antagonist MIA-690 and the EGFR inhibitor Gefitinib. Both compounds show a favourable effect in combination, decreasing cell viability and adhesion in CRPC tumour lines. In addition, our work was complemented by preliminary studies showing that MIA-690 and Gefitinib in combination can reduce the expression of metastatic factors such as β-catenin and MMPs 2 and 9, as well as cause cell cycle arrest. More trials would provide greater confidence in our hypothesis. In conclusion, combination therapy between the GHRH-R antagonist and the EGFR inhibitor could act synergistically in prostate tumour cells, blocking tumour processes.

## 4. Material and Methods

### 4.1. Peptides

Gefitinib was kindly provided by Biorbyt (Cambridge, UK). The GHRH antagonist MIA-690 was synthesized in the laboratory of Prof. Andrew Viktor Schally by solid-phase peptide synthesis and tertbutoxycarbonyl(Boc)-chemistry as previously reported. MIA-690 and Gefitinib were dissolved in 0.1% dimethyl sulfoxide (DMSO) in 10% (*vol*/*vol*) aqueous propylene glycol solution (vehicle solution). 

### 4.2. Cell Culture

A human hormone-refractory prostate cancer cell line, PC-3 (passages 7–17, ATCC CRL-1435) (RRID: CVCL_0035), representing a more aggressive stage of prostate cancer, was obtained from the American Type Culture Collection. The cell line was authenticated using short tandem repeat (STR) profiling within the past three years. PC-3 cells were cultured and maintained in RPMI-1640 medium supplemented with 10% fetal bovine serum (FBS) and 1% penicillin/streptomycin/amphotericin B (Life Technologies, Carlsbad, CA, USA). Culturing was conducted in a humidified 5% CO_2_ environment at 37 °C. Once the cells reached 70–80% confluence, they were washed with phosphate-buffered saline (PBS), detached using 0.25% trypsin/0.2% ethylenediaminetetraacetic acid (EDTA), and seeded at a density of 30,000–40,000 cells/cm². All experiments were carried out with mycoplasma-free cells, and the culture medium was replaced every 3 days.

### 4.3. Cell Viability Studies

PC-3 cells were cultured to 70–80% confluence, harvested using a trypsin/EDTA solution, and seeded at a low density (15,000 cells per well) in 96-well plates for 24 h. The culture medium was then replaced with RPMI-1640 medium containing 1% antibiotic/antimycotic solution (penicillin/streptomycin/amphotericin B) and 0% FBS for an additional 24 h. Cells were subsequently treated for 24 h with 0.1 μM MIA-690 and/or 200 μM Gefitinib. Cell viability was assessed using a tetrazolium assay, which measures the reduction of the substrate MTT [3-(4,5-dimethylthiazol-2-yl)-2,5-diphenyltetrazolium bromide] to a dark blue formazan product by mitochondrial dehydrogenases in viable cells. MTT (0.3 mg/mL) (Sigma–Aldrich, Alcobendas, Madrid, Spain) was added to each well, and the plates were incubated for 90 min at 37 °C in darkness. The medium was then removed, and the dark blue formazan precipitate was dissolved in dimethyl sulfoxide (DMSO). Absorbance was measured at 595 nm using a plate reader (ELX 800, Bio-Tek Instruments, Winooski, VT, USA). Results were expressed as a percentage of absorbance relative to control cells.

### 4.4. Cell Adhesion Assay

A concentrated stock solution of type I collagen was diluted with 10 mM glacial acetic acid and used to coat 96-well plates for 1 h at 37 °C. The plates were then washed twice with PBS (pH 7.4). Cells were harvested using 0.25% trypsin/0.2% EDTA and collected by centrifugation. Subsequently, the cells were resuspended in RPMI-1640 medium at a concentration of 2.5 × 10⁴ cells per well. After plating, non-adherent cells were removed at the specified time points by aspiration. Cell adhesion was quantified by adding an MTT solution (1 mg/mL) for a 90 min incubation. To dissolve the formazan precipitate, 100 µL of DMSO was added to each well, and absorbance was measured at 595 nm.

### 4.5. Protein Isolation and Western Blotting

PC-3 cells (3 × 10⁵ cells/well in P-6 culture dishes) were incubated with MIA-690, Gefitinib, or their combination for 24 h. After incubation, the cells were washed twice with ice-cold PBS, harvested, scraped into ice-cold PBS, and pelleted by centrifugation at 500× *g* for 5 min at 4 °C. The cells were then kept on ice for 30 min in a solution containing 20 mM Tris–HCl (pH 7.5), 1 mM EDTA, 0.5 M NaCl, 0.5% Nonidet NP-40, 2 mM phenylmethylsulfonyl fluoride (PMSF), 5 μg/mL leupeptin, 5 μg/mL aprotinin, and 5 μg/mL pepstatin. Subsequently, the cells were pelleted by centrifugation at 4000× *g* for 5 min at 4 °C. The cell lysates were then resolved on a 10% SDS-PAGE gel and transferred onto a nitrocellulose membrane (BioTrace/NT, Pall, East Hills, NY, USA) overnight in 50 mM Tris-HCl, 380 mM glycine, 0.1% SDS, and 20% methanol. The membrane was then incubated overnight at 4 °C with primary antibodies against β-catenin (1:1000) and β-actin (1:20,000). After washing with PBS, the membrane was incubated with horseradish peroxidase-conjugated anti-mouse secondary antibody (1:5000) for 1 h at room temperature. Finally, the membrane was incubated with a 1:100 solution of ECL (Luminol) and H₂O₂ for 5 min to detect the chemiluminescent signal and exposed to photographic film (AGFA). Densitometric analysis of the bands was performed using ImageJ, with results expressed as the relative percentage of intensity compared to the control, using β-actin as a positive control.

### 4.6. Gelatin Zymography

PC-3 cells (3 × 10^5^) were seeded and the different treatments—control, MIA-690, Gefitinib, or the combination of both—were performed for 24 h. The extracellular medium was collected and stored at -80 ℃ until use. To visualize the enzymatic activity of gelatinases, a 10% SDS-PAGE electrophoresis gel was run, co-polymerised with 0.1% gelatin, and maintained at 110 V at 4 ℃. Afterwards, four washes of the gel were per-formed to remove SDS: two washes of 20 min each with 50 mM Tris-HCl buffer (pH 7.4) and 2.5% Triton X-100 and two washes of 10 min each with 50 mM Tris-HCl buffer. Then, the gel was incubated overnight at 37 ℃ with buffer containing 50 mM Tris-HCl (pH 7, 4), 10 mM CaCl2, 0.15 M NaCl, 0.1% Triton X-100, and 0.02% sodium azide. Finally, the gels were stained for 30 min with 0.25% Coomassie blue R-250 and destained with a solution containing 7.5% acetic acid and 20% methanol. This resulted in the appearance of white bands on the blue background of the gel due to gelatine degradation. Densitometric analysis of metalloprotease 2 (MMP2) and metalloprotease 9 (MMP9) activity was performed with Image Lab software Version 6.1 (Bio-Rad, Hercules, CA, USA).

### 4.7. Cell Cycle Analysis

PC-3 cells (1 × 10^5^) were cultured in 6-well plates. After 24 h, the culture medium was replaced with RPMI-1640 medium containing 0% FBS and 1% antibiotic/antimycotic (penicillin/streptomycin/amphotericin B) for another 24 h. Following this, the cells were subjected to various treatments (MIA-690, Gefitinib, or their combination) for 24 h. The cells were then washed with PBS and detached using 0.25% trypsin/0.2% EDTA. Afterward, the cells were centrifuged at 500× *g* for 5 min at 4 °C, and the pellets were resuspended in ice-cold 70% ethanol and stored at −20 °C for 30 min. Following ethanol removal by centrifugation, the pellets were washed with PBS and centrifuged again. The supernatants were discarded, and the pellets were resuspended in PBS containing 100 μg/mL RNase A and 1 μg/mL propidium iodide (PI) prior to flow cytometry analysis using a MACSQuant^®^ Analyzer 10. The percentage of cells in each phase of the cell cycle was determined using MACSQuantify 2.13.2 software. A total of 10,000 cells in the selected regions were analyzed.

### 4.8. Animals, Xenografts, and Processing of Tumours

Athymic male nude mice (nu/nu) that were 5–6 weeks old were obtained from Harlan (Oxon, UK) and maintained in microisolator units on a standard sterilizable diet. Mice were housed under humidity- and temperature-controlled conditions, and the light/dark cycle was set at 12 h intervals.

For the preparation of xenografts, PC-3 cells were washed with PBS, detached with 25% trypsin/0.2% EDTA, centrifuged at 400× *g*, and suspended in fresh medium at 5 × 10^7^ cells/mL. The cell suspension was mixed with Matrigel (Becton Dickinson, Madrid, Spain) synthetic basement membrane (1:1, *v*/*v*) and then injected subcutaneously into the right flank of nude mice (5 × 10^6^ cells/mouse). Tumour volume (length × width × height × 0.5236) [28] and body weight were measured twice a week. The experiment was started when the tumours had grown to ~85 mm^3^. Animals were randomly divided into three treatment groups: group 1 (six mice), control, vehicle solution; group 2 (nine mice), MIA-690 injected subcutaneously once a day at a dose of 5 μg/animal; group 3 (nine mice), Gefitinib, intraperitoneally injected five days per week at a dose of 100 mg/kg/day; and group 4 (nine mice), both compounds. The experiment was ended on day 36. Mice were anesthetized with halothane and sacrificed. Afterwards, tumours were dissected, cleaned, and weighed. Tumour specimens were processed for immunohistochemistry (fixed in 10% formalin and paraffin-embedded tissue), and the other portions were maintained at −80 °C for further experiments.

### 4.9. In Vivo Animal Imaging 

Animals were fasted 6 h prior to the imaging assay. All mice were anesthetized with 2% isoflurane throughout all procedures. For all imaging experiments, 15 nmol of the fluorescent contrast agent, IRDye 800CW 2-DG (Deoxyglucose, LI-COR), was injected intravenously through the tail vein in order to detect PC-3 tumours [29]. Images were obtained and analyzed with an Odyssey Imaging System (LI-COR). As an autofluorescence control, a mouse without dye was scanned. At the experimental endpoints, the mice were sacrificed.

### 4.10. Determination of VEGF

VEGF levels were determined in tumour homogenates (15 µg) by ELISA (human VEGF DuoSet, R&D Systems, Madrid, Spain) according to the manufacturer’s instructions. Data were normalized to the protein concentration in each sample.

### 4.11. Histological Assays

Serial 5 μm thick tissue sections were deparaffinized in xylene and rehydrated through graded ethanol concentrations. For Masson’s Trichrome staining, the sections were first treated with Weigert’s iron hematoxylin for 5 min, followed by a 4 min wash in water. The sections were then stained with 33% acid fuchsin and 66% Ponceau for 8 min and rinsed in water containing 1% acetic acid. Subsequently, the sections were immersed in an Orange G solution for 5 min, followed by another rinse in water with 1% acetic acid, and then stained in a light green solution (0.2% light green and 0.2% acetic acid) for 3 min, with a final wash. The sections were dehydrated and mounted in Entellan^®^ for further observation.

For immunohistochemistry assays, the sections were rehydrated, placed in a glass jar containing 10 mM sodium citrate buffer (pH 6.0), and heated in a pressure cooker for 2 min. Endogenous peroxidase activity was inhibited by incubating the sections in 3% hydrogen peroxide for 20 min at room temperature. After rinsing in Tris-buffered saline (TBS), the slides were incubated with a blocking solution (3% normal donkey serum with 0.05% Triton in TBS) for 45 min to prevent non-specific binding of the primary antibody. The sections were then incubated overnight at 4 °C with primary antibodies (GHRH 1:500, GHRH-R 1:1000, pEGFR 1:100, pHER2 1:200, MMP2 1:200, MMP9 1:100) diluted 1:9 in blocking solution. Following TBS washes, the sections were incubated with the primary antibody amplifier Quanto (Ultravision Quanto detection system-peroxidase, Master Diagnostica, Granada, Spain) for 10 min. After an additional TBS wash, the sections were incubated with polymer Quanto for 10 min. Peroxidase activity was detected using a DAB kit (Master Diagnostica). The sections were then dehydrated, cleared in xylene, and mounted in Entellan^®^. To verify immunoreaction specificity, both negative and positive controls were used. Negative controls included sections processed identically but without primary antibody incubation, while positive controls consisted of skin, rat adrenal gland, and kidney sections processed with the same antibodies.

Immunoreactivity in each area of interest was quantified using stereological software, Motic Images Advanced 3.2 (Motic China Group Co., Ltd., Kowloon, Hong Kong). This software allows systematic random sampling of selected fields based on a specified sampling fraction. An average of 10 microscopic fields per section was scanned at ×20 magnification. Staining intensity was measured on a grey level scale (0: white; 255: black) using a negative image.

### 4.12. Data Analysis

Quantification of band densities was performed using Quantified One Program (Bio-Rad, Alcobendas, Spain). Data shown in the figures are representative of at least three different experiments. The results are expressed as the mean ± S.E.M. and were evaluated statistically with the Bonferroni test for multiple comparisons after one- or two-way analysis of variance (ANOVA). The level of significance was set at *p* < 0.05.

## 5. Conclusions

The importance of the results analyzed in this work led us to design an in vivo study in PC-3 tumours on the combined effect of the GHRH antagonist MIA-690 and the reversible EGFR inhibitor Gefitinib, a chemotherapeutic agent approved for clinical use in lung cancer. Combined treatment with both agents resulted in both a greater reduction in tumour growth and a significant increase in the time it took for tumour size to double compared to the effects observed with each of the compounds used individually. In addition, the analysis of the angiogenic network in the tumours generated shows that the combination of both compounds potentiated the decrease in the proangiogenic factor VEGF. On the other hand, the study of tumour glycolytic activity in the animals showed a reduced presence of metastatic foci in the group treated with the combination of both compounds.

The absence of expression of GHRH and its receptors in PC-3 tumours after combined treatment may suggest that the observed reduction in tumour growth is a consequence of the modification of the molecular machinery involved in the processes of tumourigenesis, angiogenesis, and metastasis.

## Data Availability

All figures and data are included in the manuscript.

## Abbreviations

ADT	Androgen-deprivation therapy
CRPC	Castration-resistant prostate cancer
DMSO	Dimethyl sulfoxide
EGFR	Epidermal growth factor receptors
EMT	Epithelial–mesenchymal transition
EDTA	Ethylenediaminetetraacetic acid
FBS	Fetal bovine serum
FGFR	Fibroblast growth factor receptor
GH	Growth hormone
GHRH	Growth hormone-releasing hormone
GHRH-R	Growth hormone-releasing hormone receptor
GPCRs	G protein-coupled receptors
ICAM-1	Intercellular adhesion molecule-1
IGF-I	Insulin-like growth factor type I
MMPs	Matrix metalloproteinases
MTT	3-(4,5-dimethylthiazol-2-yl)-2,5-diphenyl-tetrazole bromide
PBS	Phosphate-buffered saline
PCa	Prostate cancer
pGHRH-R	Pituitary-type growth hormone-releasing hormone receptor
PI	Propidium iodide
RTK	Receptor tyrosine kinase
TBS	Tris-buffered saline
TKIs	Tyrosine kinase inhibitors
TMPS	Triple-membrane-passing-signal
